# Leveraging GIS-based multi-criteria analysis for flood-prone area detection in Nsanje District, Malawi

**DOI:** 10.1371/journal.pone.0358998

**Published:** 2026-09-24

**Authors:** Charles Bakolo, Lusungu Ngwira, Kennedy Nazombe, Veronica Immaculate Bukani, Ken Chapita, Devie Chilonga, Harineck Mayamiko Tholo, Chikondi Chisenga, Jabulani Nyengere

**Affiliations:** 1 Department of Surveys, Ministry of Lands, City Centre, Lilongwe, Malawi; 2 Innex Consultants, Department of Research, Blantyre, Malawi; 3 Department of Land and Water Resources, Faculty of Life Sciences and Natural Resources, Lilongwe University of Agriculture and Natural Resources, Lilongwe, Malawi; 4 The Catholic University of Malawi, Department of Geography and Environmental Studies, Nguludi, Malawi; 5 Ndata School of Climate and Earth Sciences, Malawi University of Science and Technology, Limbe, Malawi; Shiv Nadar University - Campus Delhi NCR: Shiv Nadar University, INDIA

## Abstract

Flooding remains one of the most severe and recurrent natural hazards in sub-Saharan Africa, posing persistent threats to rural livelihoods, infrastructure, and environmental stability. In Malawi, the Lower Shire River Valley, particularly Nsanje district, is highly vulnerable to recurrent flooding due to climatic variability, land-use change, and geomorphological characteristics. Despite the growing availability of geospatial data, localized flood vulnerability assessments remain limited, constraining effective disaster preparedness and land-use planning. This study applied a Geographic Information System-based multi-criteria analysis using the Analytical Hierarchy Process to identify flood-prone areas in Nsanje district, Malawi. Five key spatial parameters were integrated: land cover, precipitation, slope, proximity to water bodies, and elevation. The datasets included Sentinel-2 imagery, CHIRPS precipitation records, Shuttle Radar Topography Mission digital elevation data, and official hydrological data. The weighted overlay analysis revealed that land cover and precipitation were the dominant determinants of flood susceptibility, accounting for 59.4 percent and 21.0 percent of the total influence, respectively. The resulting flood susceptibility map showed a pronounced north–south gradient in vulnerability, with the southern corridor exhibiting the highest exposure to flooding. Traditional Authority Nyachikadza emerged as the most critically affected area, with more than 1,500 residents situated in high-risk zones, whereas Traditional Authorities Malemia and Tengani displayed moderate levels of vulnerability. The findings indicate that the combined effects of land-use practices, rainfall intensity, and terrain configuration strongly influence flood susceptibility in Nsanje. This study provides a cost-effective and replicable framework for flood risk assessment in data-scarce environments. By leveraging freely available satellite data and open-source geospatial tools, it offers actionable intelligence to support disaster management, spatial planning, and community resilience in flood-prone areas. This approach contributes to the broader sustainability agenda by informing climate adaptation strategies that are aligned with national and global commitments to disaster risk reduction and Sustainable Development Goals. The five-factor model used here should be read as a first-order, operationally feasible approximation of flood susceptibility; geology, soil permeability, and drainage density were not incorporated and are discussed as an explicit limitation, and the precipitation criterion reflects cumulative seasonal totals rather than storm-scale rainfall intensity.

## 1. Introduction

Flooding is one of the most recurrent natural hazards in sub-Saharan Africa, causing widespread disruption to livelihoods, infrastructure, and ecosystems in the region. In Malawi, floods represent a persistent environmental and socio-economic challenge and rank among the most frequent disasters affecting rural communities [1]. The country’s geographic setting, characterized by extensive river basins and low-lying flood plains, contributes to high flood susceptibility, which is further influenced by climate variability and land-use change [2,3]. The Lower Shire River Valley in southern Malawi exemplifies this condition, experiencing recurrent flooding driven by intense rainfall, river overflow, and limited drainage capacity [4]. Within this valley, Nsanje district is particularly affected, with repeated inundation disrupting agricultural production, displacing populations, and damaging infrastructure [5].

Recent flood intensification in Malawi has been associated with land-use transformation and climate change. Deforestation, agricultural expansion, and settlement encroachment into floodplains have reduced natural water retention capacity and increased surface runoff [6,7]. Simultaneously, changes in rainfall regimes associated with climate change have increased the frequency of extreme precipitation events [8]. These processes place additional pressure on floodplain-dependent communities, particularly those that rely on rain-fed agriculture. The 2019 flooding associated with Tropical Cyclone Idai, which displaced nearly 87,000 people in Nsanje district, illustrates the scale of flood impacts in the region [9]. Despite the recurrence of such events, localized flood risk assessments supporting district-level planning remain limited.

Flood assessment efforts in Malawi have traditionally relied on hydrological and hydraulic modeling approaches that require dense observational datasets, detailed channel geometry, and substantial technical capacity. In data-scarce and resource-constrained settings, these requirements limit their applicability for routine district-scale analysis [10,11]. As a result, planners and disaster management agencies often lack spatially detailed information to support preparedness and mitigation activities. Advances in geospatial technology have provided alternative approaches. Geographic Information Systems (GIS), combined with multi-criteria decision-making methods such as the Analytical Hierarchy Process (AHP), allow the integration of environmental, climatic, and topographic variables for flood susceptibility mapping in a transparent and cost-effective manner [12,13].

Although GIS-AHP approaches have been applied in flood susceptibility studies globally [14–16], their applications in Malawi remain limited, particularly at fine administrative scales. Previous studies have largely focused on basin or national-level assessments, which may obscure spatial variability in flood exposure at the Traditional Authority (TA) level [2,6]. The use of freely available satellite datasets, including Sentinel-2 land cover imagery, CHIRPS precipitation data, and Shuttle Radar Topography Mission (SRTM) elevation products, provides an opportunity to improve flood susceptibility mapping in data-scarce environments [17,18]. These datasets enable the production of spatially detailed flood maps suitable for operational planning. Recent research further supports the integration of open-access earth observation datasets with MCDA techniques for flood susceptibility mapping, particularly in developing regions where monitoring infrastructure is sparse [19–21]. The use of Sentinel-derived land cover products, CHIRPS precipitation datasets, and SRTM-derived elevation models has been shown to improve reproducibility and scalability of susceptibility assessments while maintaining methodological transparency [22,23].

Although GIS–AHP approaches have been widely applied in flood susceptibility studies globally [14–16], recent studies demonstrate continued methodological refinement and expanded regional applications. For example, GIS-based MCDA and AHP frameworks have been successfully applied in diverse climatic and geomorphological settings, including rapidly urbanizing basins and semi-arid environments, to improve spatial flood prediction accuracy and planning relevance [19,20,22–26]. Recent applications integrating remote sensing datasets and hybrid GIS–MCDA models highlight their operational suitability in data-scarce regions similar to sub-Saharan Africa [21,27,28]. These studies confirm that index-based and multi-criteria approaches remain highly adaptable for district- and sub-district-scale flood susceptibility assessments where hydrometric records are limited.

Second, the integration of freely available satellite datasets, including Sentinel-2 land cover imagery, CHIRPS daily precipitation records, and SRTM elevation products, offers a replicable and cost-effective approach for resource-constrained districts in sub-Saharan Africa. Unlike previous studies that relied on proprietary datasets or intensive field campaigns, this methodology can be readily adapted by local government technical staff with basic GIS training and access to open-source software applications. The validation of the results using documented flood events from 2015 and 2019, including the impacts of Tropical Cyclone Idai, provides an empirical grounding that strengthens confidence in the approach.

Third, compared to alternative flood assessment methodologies, the GIS–AHP approach was selected as the most appropriate method given the specific constraints and requirements of the Nsanje District context. Hydrodynamic models, while providing detailed inundation predictions, require extensive input data, including channel cross-sections, Manning’s roughness coefficients, detailed bathymetry, and calibrated discharge time series, none of which are systematically available for Nsanje’s river network. Machine learning approaches, including random forests and neural networks, depend on comprehensive historical flood inventories with spatially explicit inundation extents and dates. In Nsanje, historical flood documentation consists primarily of qualitative reports and displacement statistics from Traditional Authorities, without the georeferenced inundation boundaries necessary for model training and validation. Probabilistic methods based on frequency analysis require long-term hydrometric records from multiple gauging stations; however, the Lower Shire Valley has limited functional stream gauges, and existing records contain significant gaps due to equipment failures and maintenance challenges.

Within these constraints, the GIS-AHP approach provides a transparent, stakeholder-inclusive, and operationally feasible means of integrating available spatial datasets to support flood risk assessment and planning. The participatory weighting process allows for the incorporation of expert knowledge and local understanding of flood-generating processes, compensating for data limitations through a structured judgment. While this introduces an element of subjectivity, recognized as a limitation discussed in Section 4, the consistency ratio verification ensures logical coherence in the weighting process [29]. Recent methodological advancements demonstrate the continued relevance of AHP within GIS-based flood susceptibility modeling frameworks. Comparative studies evaluating AHP against machine learning and hybrid statistical approaches indicate that, despite its subjective weighting structure, AHP performs competitively when data availability is constrained and expert knowledge is essential [24–26]. Furthermore, the consistency ratio verification process enhances logical coherence and reliability in spatial multi-criteria weighting [20,29]. Recent sustainability-focused applications also highlight the utility of AHP for supporting climate adaptation and disaster risk reduction planning at local governance scales [27,28].

Therefore, the novelty of this study lies not in the development of new analytical techniques but in the strategic adaptation and validation of established methods to meet the specific needs of a data-limited but disaster-vulnerable district in southern Malawi. The transferable insights from this application include the following: (1) demonstration that sub-district flood mapping is achievable using open-access satellite data in regions with minimal ground-based monitoring infrastructure; (2) validation protocols that leverage event-based documentation even in the absence of continuous monitoring; and (3) a workflow that can be institutionalized within district planning departments to support evidence-based land-use zoning and disaster preparedness. These contributions directly address the global challenge of bridging the gap between sophisticated flood modeling capabilities and the operational realities faced by local governments in developing countries.

This study applies a GIS-based multi-criteria analytical framework to identify flood-prone areas in Nsanje District, Malawi. The analysis integrates spatial datasets representing key flood-influencing factors, including land cover, slope, precipitation, elevation, and proximity to water bodies. The Analytical Hierarchy Process is used to derive relative weights for these factors, and the resulting flood susceptibility map is produced at the Traditional Authority level to support disaster preparedness and land-use planning. The approach is designed to be replicable and applicable in other data-limited floodplain environments.

This study contributes empirical spatial information relevant to flood risk management and sustainable development planning. Flooding poses challenges to food security, infrastructure resilience, and social well-being, which are central concerns within the United Nations Sustainable Development Goals, particularly Goals 11 (Sustainable Cities and Communities) and 13 (Climate Action) [30]. By providing spatially explicit flood susceptibility information, this study supports evidence-based decision-making by local authorities and development stakeholders.

Although GIS-AHP methods are well established, this study extends their application by implementing Traditional Authority-level flood susceptibility mapping in Malawi’s Lower Shire Valley, integrating freely available satellite data, and validating the results using flood events from 2015 and 2019. The GIS–AHP approach was selected over hydrodynamic, machine learning, and probabilistic models due to the limited availability of data, historical flood records, and hydrometeorological observations in Nsanje District. Within these constraints, the approach provides a transparent and operationally feasible means of supporting flood risk assessment and planning.

## 2. Materials and methodology

### 2.1. Study area

**Fig 1:**
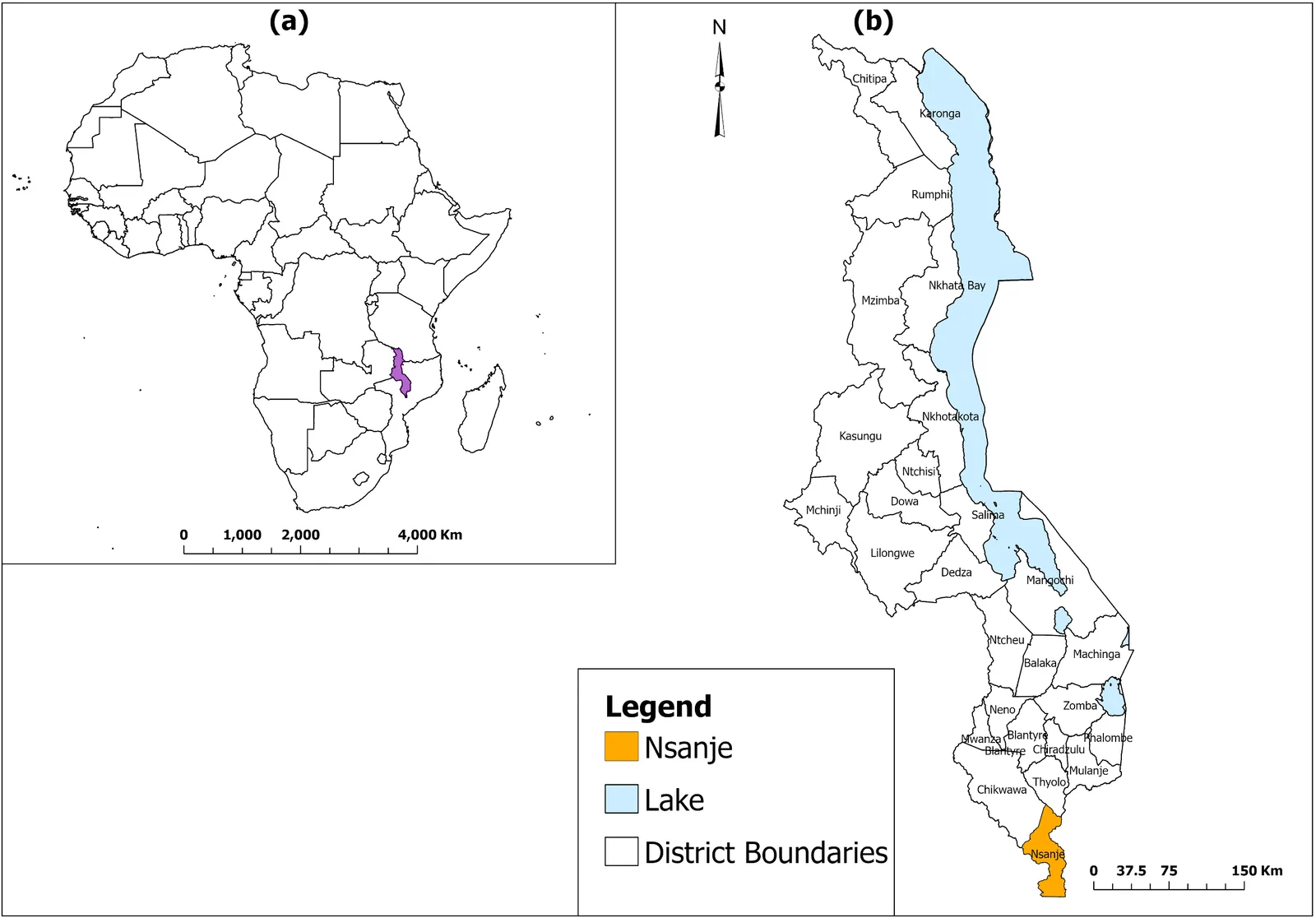
The inset map of Africa shows the location of Malawi on the continent (a). Map of Malawi showing Nsanje district, the study area (b). Data Source: Malawi National Statistics Office & the Department of Surveys and Mapping (2020).

This study was conducted in Nsanje district (Fig 1), located in the Southern Region of Malawi, between latitudes 16°45′00″S and longitudes 35°10′00″E. The district covers approximately 1,942 km^2^ and has an estimated population of about 300,000 people [31]. Situated in the Lower Shire River Valley along the border with Mozambique, Nsanje is Malawi’s southernmost district. It lies between 30 and 602 m above sea level, with most areas between 30 and 206 m [32]. Nsanje serves as an important administrative, trade, and transport hub due to its strategic position along the Shire River and its proximity to Mozambique.

The district’s economy is predominantly agrarian, with rainfed agriculture serving as the principal livelihood strategy. Major crops include maize, millet, sorghum, rice, sweet potatoes, beans, and cotton, while livestock production, mainly cattle, goats, sheep, and poultry, provides supplementary income [33]. The climate of Nsanje is characterized by distinct seasonal variability, with mean annual temperatures ranging from 19.4°C to 36.1°C and average annual precipitation between 800 mm and 1,200 mm [34].

Nsanje district faces significant environmental and climatic challenges due to its location in the Lower Shire Valley. The area is highly vulnerable to extreme weather events, including recurrent droughts and floods, which adversely affect agricultural productivity and livelihoods. These climatic shocks, coupled with poor natural resource management and high population pressure, have exacerbated land degradation, deforestation, and loss of natural vegetation cover [33,35]. Consequently, the district experiences persistent socio-economic and environmental vulnerabilities that undermine sustainable development efforts.

### 2.2 Research design

**Fig 2:**
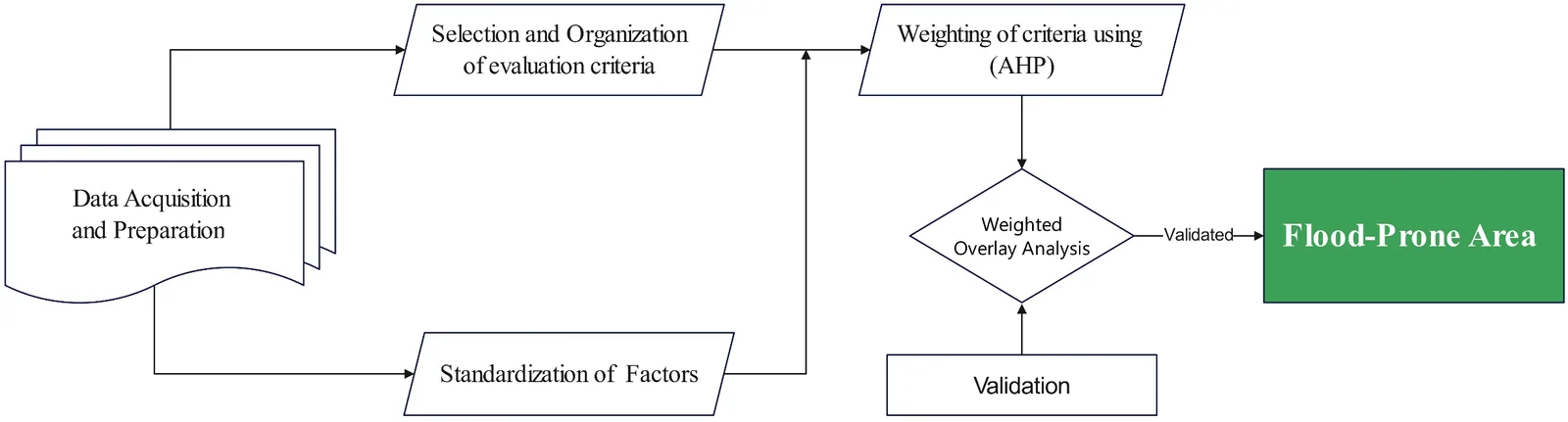
Research design.

This study employed a spatial Multi-Criteria Evaluation (MCE) approach that integrates geospatial analysis and decision-support techniques to assess site suitability in a systematic and reproducible manner. The overall methodological framework (Fig 2) was structured into six sequential phases: data acquisition and preparation, selection and organization of evaluation criteria, standardization of factors, weighting of criteria using the Analytical Hierarchy Process (AHP), weighted overlay analysis, and validation of the final outputs. This design ensured consistency, transparency, and analytical rigor throughout the assessment process. Each component of the framework is described in detail in the subsequent sections.

### 2.3. Data acquisition and preparation

To ensure the reliability and accuracy of subsequent spatial and statistical analyses, multiple datasets (Table 1) were obtained from primary and secondary sources to characterize the geographic and environmental characteristics of Nsanje district. The acquired data underwent a systematic preprocessing workflow, including validation, cleaning, projection harmonization, and integration within a geospatial framework to facilitate effective analysis.

**Table 1:** Spatial datasets and their corresponding analysis tools.

No.	Factors	Criteria	Source	Analysis tool
1	Geographic	30 m resolution DEM in raster format	USGS SRTM 1 Arc-Second Global DEM. Retrieved from Earth Explorer (earthexplorer.usgs.gov). Open Access Data.	Reclassify
		30 m resolution Slope in raster format generated from the DEM	Derived from USGS SRTM 1 Arc-Second Global DEM using ArcGIS Spatial Analyst tools. Open Access Data.	Reclassify
		Land cover	10 m spatial resolution Sentinel-2 image from Copernicus Global Land Cover, MODIS Land Cover. Open Access Data.	Reclassify
2	Environmental	Average precipitation (2024/2025 season)	CHIRPS Daily Precipitation Data WorldClim-Open Access Data.	Reclassify
		Proximity to Water bodies	Malawi National Statistics Office & the Department of Surveys and Mapping (2020).	Multiple Ring Buffer

#### 2.3.1 Defining and assigning ratings to criteria and sub-criteria.

To identify flood-prone areas in Nsanje district, Malawi, a structured approach was employed to evaluate and assign ratings to criteria and sub-criteria based on their contributions to flood susceptibility [36,37]. This methodology provided a robust foundation for effective flood risk assessment and decision-making protocols (Table 2).

**Table 2:** Standardized scores for criteria and sub-criteria.

Criteria used	Sub-criteria	Standardization score	Factor suitability rating
**Precipitation (mm)**	1–315	1	Unsuitable
	315-828	2	Poorly suitable
	828-1287	3	Moderate suitable
	1287-1906	4	Suitable
	1906–3843	5	Highly suitable

**Digital elevation model (DEM) in metres**	19–611	5	Highly suitable
	611–1203	4	Suitable
	1203–1795	3	Moderate suitable
	1795–2387	2	Poorly suitable
	2387–2979	1	Unsuitable

**Slope (%)**	0 - 15.52	5	Highly suitable
	15.52 - 31.05	4	Suitable
	31.05 - 46.57	3	Moderate suitable
	46.57 - 62.10	2	Poorly suitable
	62.10 - 77.62	1	Unsuitable


**Proximity to water bodies (metres)**	0-250	5	Highly suitable
	250 −500	4	Suitable
	500 −1000	3	Moderate suitable
	1000 −2000	2	Poorly suitable
	≥2000	1	Unsuitable

**Land cover**	Built-up	5	Highly suitable
	Grassland/Shrubland/Herbaceous Vegetation/Bare Soil/Sparse Vegetation/Forests	4	Suitable
	Cropland	3	Moderate suitable
	Permanent Waterbodies/Herbaceous Wetland	2	Poorly suitable

The DEM has emerged as a critical factor in flood susceptibility mapping, with lower elevations considered highly susceptible to flooding owing to natural water accumulation and drainage patterns [14,38]. Areas at lower elevations are more prone to inundation during heavy rainfall events and river overflows, making elevation a fundamental criterion for flood risk assessment.

The slope gradient was identified as another crucial consideration in flood susceptibility analysis. Gentle slopes and flat terrain facilitate water accumulation and slow runoff, increasing the flood risk, whereas steeper slopes promote rapid drainage and reduce flood susceptibility [16,39]. Therefore, areas with minimal slope gradients were assigned higher flood susceptibility ratings.

Precipitation patterns are the main causes of flooding in this region. Higher precipitation intensities and accumulated rainfall increase surface runoff and the likelihood of flooding [15,40]. CHIRPS precipitation data provide crucial insights into rainfall patterns that directly influence flood occurrence and magnitude in Nsanje district.

Rainfall drives flooding through two related but distinct mechanisms: short-duration, high-intensity storm cells that generate flash runoff, and cumulative seasonal wetness that governs catchment saturation and the baseline propensity of the landscape to convert rainfall into runoff. This study represents the second of these mechanisms, using cumulative 2024/2025 seasonal CHIRPS totals as a proxy for antecedent moisture and total water input, rather than the first. This choice was made because no sub-daily or event-based rainfall intensity product with adequate spatial coverage exists for Nsanje district: the district’s automatic weather station network is sparse, and CHIRPS itself, though it can be aggregated to daily values, is a satellite-gauge blended product whose accuracy at sub-daily timescales has not been established for this region. We agree with the reviewer that this is an important simplification. Its principal implication is that the precipitation criterion in this study reflects where the landscape is wettest over a season rather than where the most intense storm cells occur in a given event, and it will therefore tend to under-represent flood susceptibility in localities that are prone to convective, flash-flood-generating storms but do not otherwise receive high seasonal totals. We consider this a plausible partial explanation for the 12 percent of the Sentinel-1-observed 2019 inundation extent that fell outside the high/very-high susceptibility classes (Section 3.4.2), and we now state this explicitly as a limitation in Section 4 and recommend that future iterations of this model incorporate intensity-duration-frequency data as soon as local station records permit.

Proximity to water bodies is considered a fundamental factor, as areas closer to rivers, streams, and other water bodies face higher flood risks owing to potential overflow and channel capacity limitations during extreme weather events [18,41]. The Shire River and its tributaries in Nsanje district are the primary sources of flood risk for the surrounding communities.

Land cover characteristics significantly influence flood susceptibility by affecting surface runoff and infiltration rates. Built-up areas and impervious surfaces increase surface runoff and flood risk, whereas vegetated areas and forests promote infiltration and reduce flood susceptibility [7,42]. Agricultural lands and bare soils pose intermediate flood risk, depending on management and seasonal conditions.

The five criteria selected for this analysis, elevation, slope, precipitation, proximity to water bodies, and land cover, represent the primary controls on flood generation and propagation in low-gradient alluvial floodplain environments such as the Lower Shire Valley. Each criterion corresponds to a specific hydrological process governing flood susceptibility in this setting.

Elevation determines gravitational potential energy and the direction of surface water flow. In floodplains, lower-elevation areas function as terminal sinks for runoff convergence and are zones where the hydraulic gradient approaches zero, resulting in reduced flow velocity and increased residence time. During flood events, these topographic lows experience the longest inundation durations and the greatest water-depth accumulation.

Slope governs flow velocity and runoff generation mechanisms. Steep slopes (>15 percent) promote rapid drainage via gravity-driven overland flow, thereby minimizing infiltration time and reducing flood accumulation. Conversely, gentle slopes (<5 percent), which characterize much of Nsanje district, facilitate water ponding, increased infiltration, and slower runoff routing to channels. In flat terrain, minor topographic variations exert disproportionate influence on inundation extent because hydraulic gradients are insufficient to drive rapid drainage.

Precipitation is the primary hydrological forcing of flood events in the Shire River Basin. The intensity and spatial distribution of rainfall control both the magnitude of direct runoff and the rate of increase in channel discharge. In the absence of dense rain gauge networks, the CHIRPS dataset provides gridded daily precipitation estimates at 0.05-degree resolution (~5 km), derived from satellite infrared cold cloud duration and calibrated against available ground observations. While CHIRPS data represent cumulative totals rather than rainfall intensity, they capture the spatial variability of seasonal precipitation patterns that drive flood occurrence. The use of cumulative precipitation for the 2024/2025 season provides a proxy for antecedent moisture conditions and total water input to the system. It is acknowledged that sub-daily rainfall intensity and storm event characteristics would provide superior hydrological discrimination; however, such data are not systematically available for Nsanje district. Future refinements of this methodology should incorporate intensity-duration-frequency relationships when local meteorological station records become accessible.

Proximity to water bodies reflects hydraulic connectivity to flood sources. The Shire River and its distributary channels represent the primary source of floodwater during high-flow events. Channel capacity is exceeded during extreme precipitation, resulting in overbank flow and lateral inundation of adjacent floodplains. Areas within 250 meters of channels experience the highest flood frequency and greatest inundation depths due to direct hydraulic connection. The buffer distance classification used in this study (250 m, 500 m, 1000 m, 2000 m, > 2000 m) reflects observed inundation extents from the 2015 and 2019 flood events as documented in post-disaster assessments [9].

Land cover influences both infiltration capacity and surface roughness, which together govern runoff generation and flow routing. Built-up areas and compacted agricultural soils exhibit low infiltration rates, resulting in a higher proportion of rainfall being converted to surface runoff. Natural vegetation, particularly forests and wetlands, promotes infiltration, increases interception storage, and retards overland flow through increased Manning’s roughness. The expansion of cropland and settlement in the Lower Shire Valley over the past two decades has systematically reduced natural water retention capacity, amplifying flood peaks and extending inundation duration [6].

### Limitations and omitted factors

It is recognised that the five-factor framework employed in this study is a simplified representation of the hydrological system. Several additional factors known to influence flood processes were not incorporated due to data availability constraints:

Geology and soil properties-Underlying geology and soil permeability control subsurface water movement and determine the partitioning between infiltration and runoff. Clay-rich soils with low hydraulic conductivity generate higher runoff coefficients than sandy soils. However, systematic soil surveys with measured hydraulic conductivity values are not available for Nsanje district. National-scale soil maps exist but lack the spatial resolution and attribute details necessary for district-level hydrological modeling.

Antecedent soil moisture-Initial soil moisture conditions before rainfall events strongly influence runoff generation. Saturated soils produce an immediate runoff response, while dry soils exhibit higher infiltration capacity. Soil moisture data would require either in-situ sensor networks or satellite-derived products with appropriate temporal resolution. Neither is systematically available for the study area.

Proximity to tectonic structures-Geological faults and fracture zones can create preferential flow paths and influence subsurface drainage patterns. However, surface processes rather than structural controls dominate the Lower Shire Valley’s flood regime, and detailed fault mapping at the required scale is not available. Drainage density and channel morphology: The configuration and capacity of the drainage network influence flood routing and storage. Detailed channel surveys would enable hydraulic capacity assessments, but such data are not available for the study area.

Excluding geology, soil permeability, and drainage density is a methodological limitation, not merely a data-availability constraint. Relevant global datasets for Nsanje District are publicly available, including SoilGrids, which provides modeled estimates of soil texture, bulk density, and derived hydraulic properties at 250 m resolution. HydroSHEDS/HydroRIVERS can derive stream networks and drainage-density metrics. These datasets were not incorporated because their suitability for representing locally variable hydrological properties in the Lower Shire Valley has not been adequately established. In particular, SoilGrids estimates are model-derived. Without locally available ground-truth hydraulic measurements, using them to represent soil permeability in an alluvial floodplain could introduce uncertainty without improving local predictive validity. Expanding the AHP framework from five to seven criteria would increase pairwise comparisons from 10 to 21. This would increase subjective judgments and potentially compound uncertainty in criterion weighting. This is especially relevant for geological and subsurface drainage controls, where locally derived empirical evidence is limited compared with the more directly observable surface characteristics represented by the current criteria (land cover, slope, proximity to water, and elevation). Accordingly, geology, soil permeability, and drainage density are treated as methodological limitations of the current framework. Future work should prioritize local validation of SoilGrids-derived soil hydraulic properties.

The decision to limit the analysis to five factors reflects a pragmatic balance between hydrological completeness and operational feasibility. The selected factors represent the primary first-order controls on flood susceptibility that can be characterized using available spatial datasets. While including additional factors would enhance model sophistication, the marginal improvement in predictive accuracy must be weighed against the costs of data acquisition and model complexity. The validation results presented in Section 3.4 suggest that the five-factor model captures the dominant spatial patterns of flood exposure, as evidenced by correspondence with documented 2015 and 2019 inundation zones.

This correspondence shows the model captures the dominant spatial pattern of exposure reasonably well. However, it does not prove that adding geology, soil, or drainage criteria would not improve model performance. The potential contribution of these variables remains an important area for further investigation.

Future research should prioritize collecting field-based measurements of soil hydraulic conductivity, establishing soil moisture monitoring networks, and conducting detailed topographic surveys of channel geometry. This data would enable the incorporation of additional factors and a transition toward more physically based hydrological modeling frameworks.

#### 2.3.2 Calculating the weights of the selected factors.

This study employed a GIS-based multi-criteria decision-making analysis, which involved assigning weights to each flood risk factor map [43]. This weighting process is crucial for expressing the relative importance of each criterion in influencing flood susceptibility and identifying flood-prone areas.

**Fig 3:**
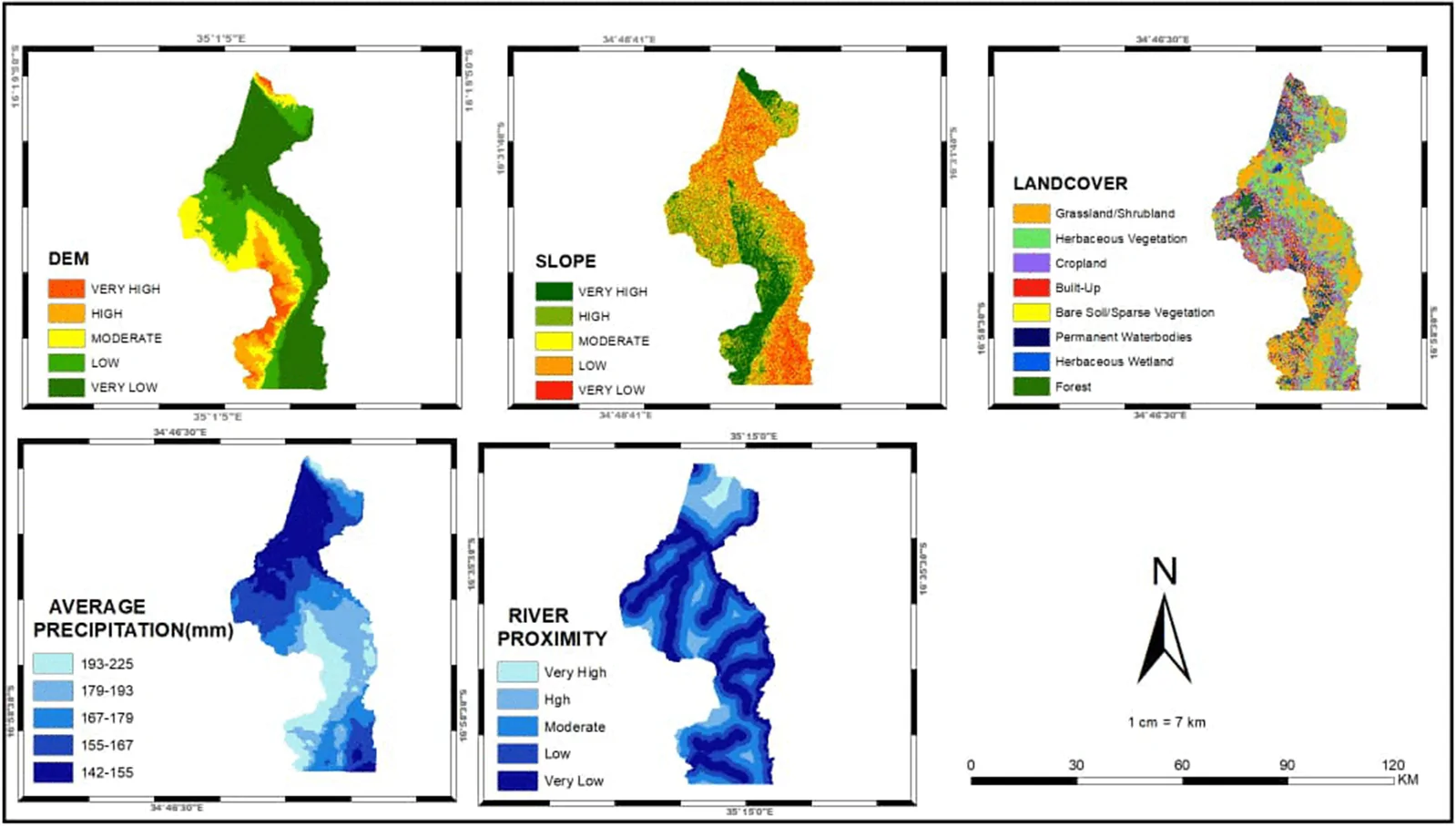
Reclassified criteria maps. Data Sources: USGS SRTM 1 Arc-Second Global DEM. Retrieved from Earth Explorer (earthexplorer.usgs.gov). Open Access Data, DUSGS SRTM 1 Arc-Second Global DEM using ArcGIS Pro 3.4 Spatial Analyst tools. Open Access Data, 10 m spatial resolution Sentinel-2 image from Copernicus Open Access Hub in Google Earth Engine (GEE). Open Access Data, CHIRPS Daily Precipitation Data-Open Access Data, and Malawi National Statistics Office & the Department of Surveys and Mapping (2020). All the data was analyzed by the Author using ArcGIS Pro 3.4.

The weights were determined using the Analytic Hierarchy Process (AHP) with pairwise comparison matrices [13,17]. Following Saaty’s [43] nine-degree preference scale (Table 3 and Fig 3), the AHP process involved pairwise comparisons of flood risk criteria, with the results entered into a comparison matrix. This hierarchy demonstrated the increasing importance of each level, thereby ensuring the incorporation of weighted criteria and facilitating flood-risk assessment and decision-making.

**Table 3:** Saaty’s (1980) Fundamental Scale for Pairwise Comparison, adapted from Gelan (2021).

Intensity of importance on an absolute scale	Definition	Explanation
**1**	Equal importance	Two activities contribute equally to the objective
		
**3**	Moderate importance of one over another	Experience and judgment strongly favour one activity over another
		
**5**	Essential or strong importance	Experience and judgment strongly favour one activity over another
		
**7**	Very strong importance	An activity is strongly favoured, and its dominance is demonstrated in practice
		
**9**	Extreme importance	The evidence favouring one activity over another is of the highest possible order of affirmation
		
**2,4,6,8**	Intermediate values between the two adjacent judgments	When compromise is needed

Saaty’s [43] consistency principle guided pairwise comparisons to ensure self-consistency in flood susceptibility evaluation. The scores reflected equal (1), high (9), or low (1/9) importance, enabling a systematic, structured approach to weighting flood risk factors by their relative contribution to flood occurrence.

This weighted multi-criteria analysis approach has been widely adopted in flood risk assessment and in decision-making processes for disaster management [12,37,44].

The criterion weights were determined by normalizing the eigenvector of the reciprocal ratio matrix. This led to the creation of a Normalized Pairwise Comparison Matrix (PCM). The standardization of these weights involved dividing each element by the total number of columns in the matrix. The following steps were used to identify the criteria weights using the AHP. From PCM, m=(n×n) for n criteria. Where ${\text{P}}_{\text{ij}}$ is the value of the cell located in the i-th row and j-th column of PCM (Eq. 1) (Eq. 1) [45].


$$\begin{array}{c} P_{ij}\;P_{ij}=\text{1} \end{array}$$ (1)

After performing pairwise comparison and calculating the factor weights using the Analytic Hierarchy Process (AHP), the consistency ratio (CR) was computed (Eq. 2). This additional step helps identify any inconsistencies in the pairwise comparisons. It determines the ideal weights for the complete pairwise comparison matrix [29,43,46].


$$\begin{array}{c} CR=\frac{CI}{RI} \end{array}$$ (2)

where CR is the Consistency Ratio, CI is the Consistency Index, and RI is the Random Inconsistency Index whose value depends on the number of factors being compared (Table 4) [43]. The consistency index (CI) (Table 5) was calculated using Eq. 3:


$$\begin{array}{c} \text{CI}=\frac{\lambda \text{max}-\text{n}}{\text{n}-\text{1}} \end{array}$$ (3)

where n = the number of items compared in the matrix, λmax = Average value of the consistency vector (Table 5).

**Table 4:** Random Inconsistency Index, where n represents the number of factors in the study, and RI denotes the Random Inconsistency Index.

N	1	2	3	4	5	6	7	8	9	10
**RI**	0	0	0.58	0.9	**1.12**	1.24	1.32	1.41	1.45	1.49

**Table 5:** Factor weight computation and consistency ratio estimation.

FACTORS	LC	P	PWB	SLP	DEM	WEIGHTS	λ	CI	RI	CR
Land cover (LC)	0.611	0.751	0.632	0.531	0.444	0.594	5.316	0.079	1.12	0.071
Precipitation (P)	0.122	0.150	0.237	0.319	0.222	0.210				
Proximity to water (PWB)	0.076	0.050	0.079	0.027	0.111	0.069				
Slope (SLP)	0.122	0.030	0.026	0.106	0.167	0.090				
Digital elevation model (DEM)	0.068	0.019	0.026	0.018	0.056	0.037				
**CHECKING**	**1.000**	**1.000**	**1.000**	**1.000**	**1.000**	**1.000**				

***LC:*** *Land cover,* ***P:*** *Precipitation,* ***PWB:*** *Proximity to water,* ***SLP****: Slope, and* ***DEM****: Digital elevation model.*

### 2.4. Data analysis

**Fig 4:**
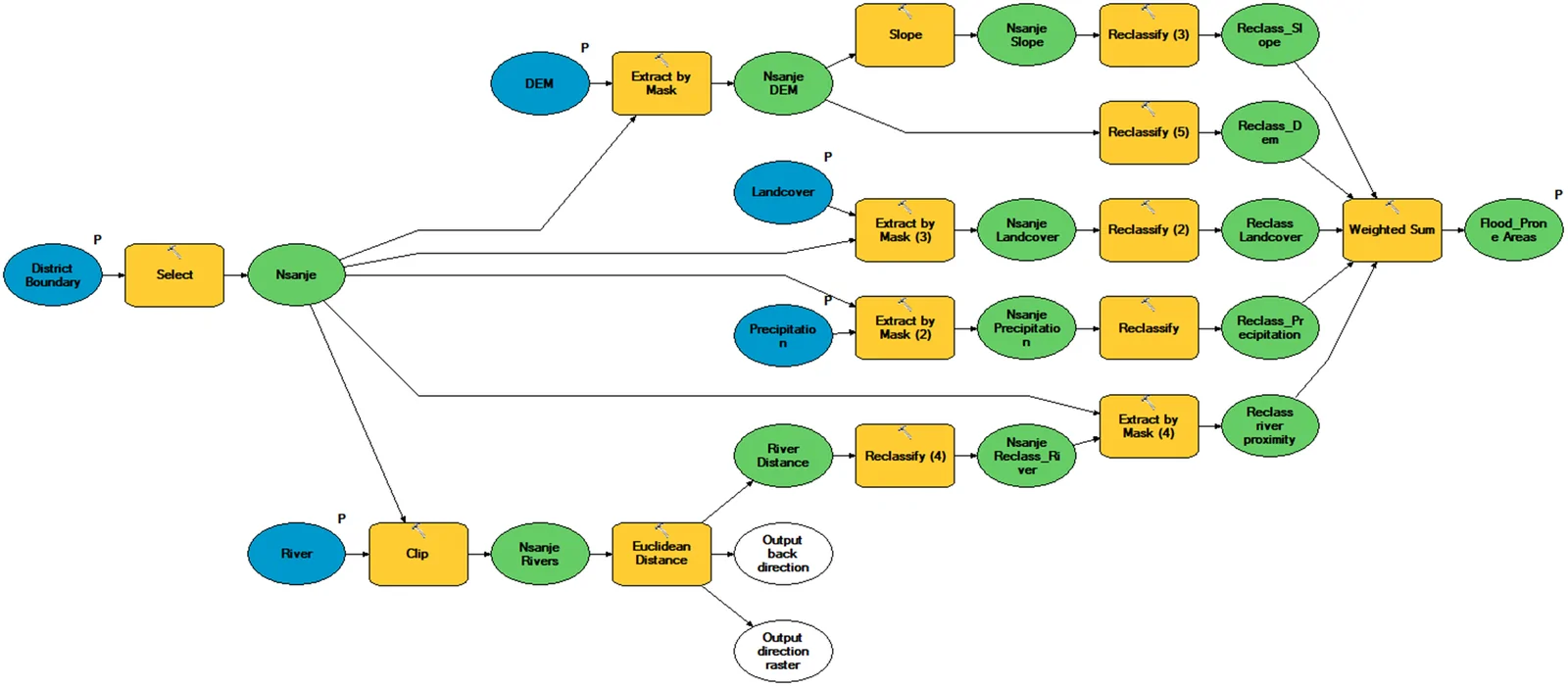
Flood-prone area detection in Nsanje district analysis workflow through ArcGIS Pro 3.3 model builder tool.

A multi-criteria analysis decision rule was applied after the weights and the reclassified criterion maps were established. Three common decision rules in multi-criteria analysis are weighted linear overlay, Boolean overlay, and ordered averaging, as noted by Jiang and Eastman [47] and Malczewski [48]. The standardized layers in this investigation were aggregated using a weighted linear combination technique in the Raster Calculator in ArcGIS Pro (Fig 4). The weight of the suitability parameters (Wi) was multiplied by factors and parameters (Xi) in the weighted linear combination technique to obtain composite weights, which were then summed [48–50] (Eq. 4).


$$\begin{array}{c} S=\sum_{i=\text{1}}^{n}\left(WiXi\right) \end{array}$$ (4)

where S = total suitability score, Wi = weight of the selected suitability criteria layer, Xi = assigned sub-criteria score of suitability criteria layer i, n = total number of suitability criteria layers.

## 3. Results

### 3.1. Criteria weighting from AHP analysis

**Fig 5:**
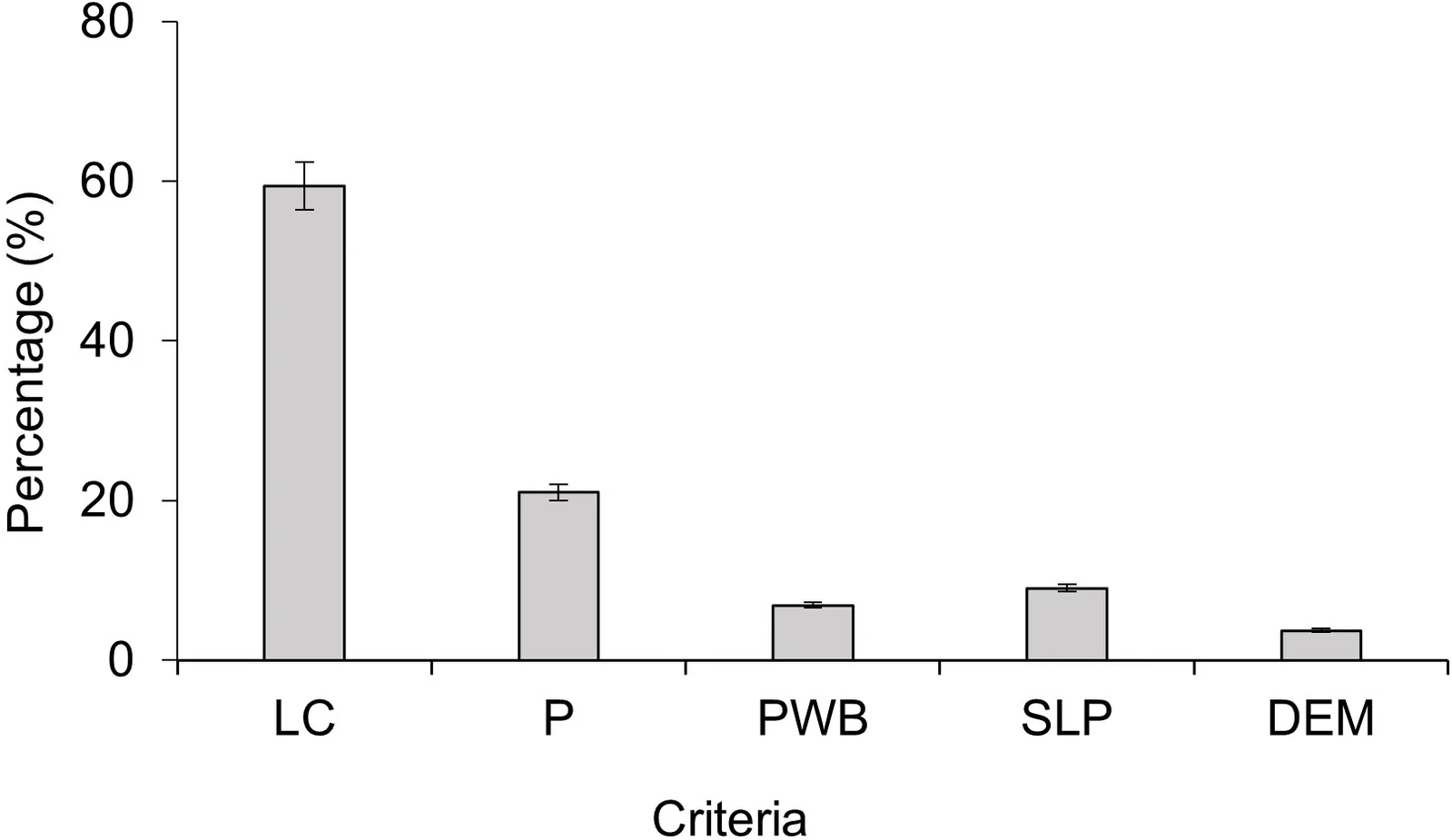
Criteria weights in percentage LC: Land cover, P: Average precipitation for 2024/2025, PWB: Proximity to water bodies, SLP: Slope, and DEM: Digital elevation model.

The AHP analysis yielded distinct importance weights for the five flood susceptibility factors (Fig 5). Land cover (LC) received the highest weight at 0.594 (59.4%), followed by Precipitation (P) at 0.210 (21.0%). Slope (SLP) ranked third with 0.090 (9.0%), and Proximity to water bodies (PWB) obtained 0.069 (6.9%). The Digital Elevation Model (DEM) received the lowest weight, 0.037 (3.7%).

### 3.2. Spatial distribution of flood-prone areas

**Fig 6:**
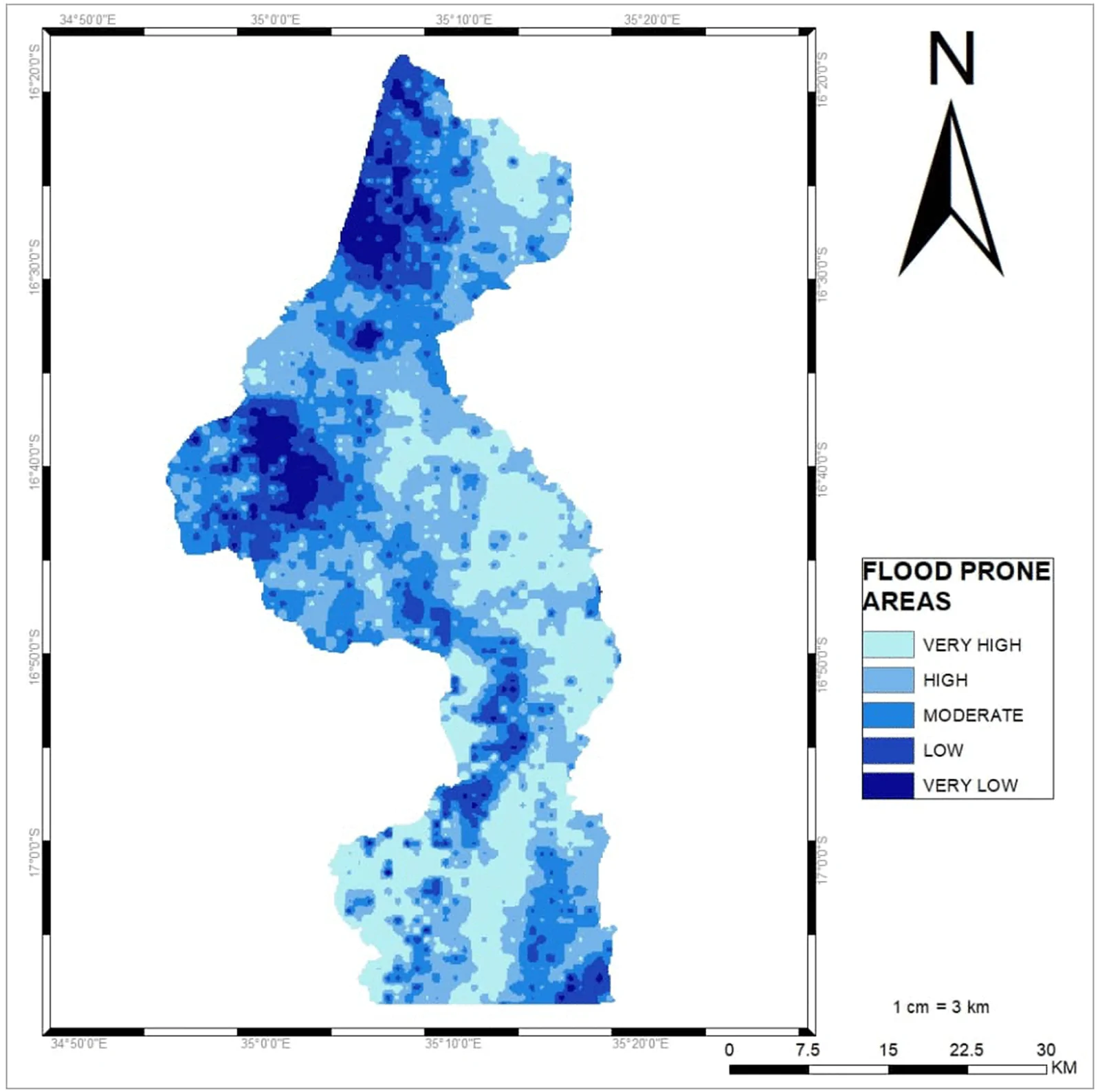
Flood-prone areas in Nsanje district.

The flood susceptibility map (Fig 6) revealed a clear north-south gradient in the risk distribution across Nsanje district. The northern and central regions were predominantly classified as low- to moderate-susceptibility zones, whereas the southern corridor exhibited concentrated areas of high- to very-high flood risk, representing approximately 30–40% of the district’s total area.

### 3.3. Traditional authority-level vulnerability assessment

**Fig 7:**
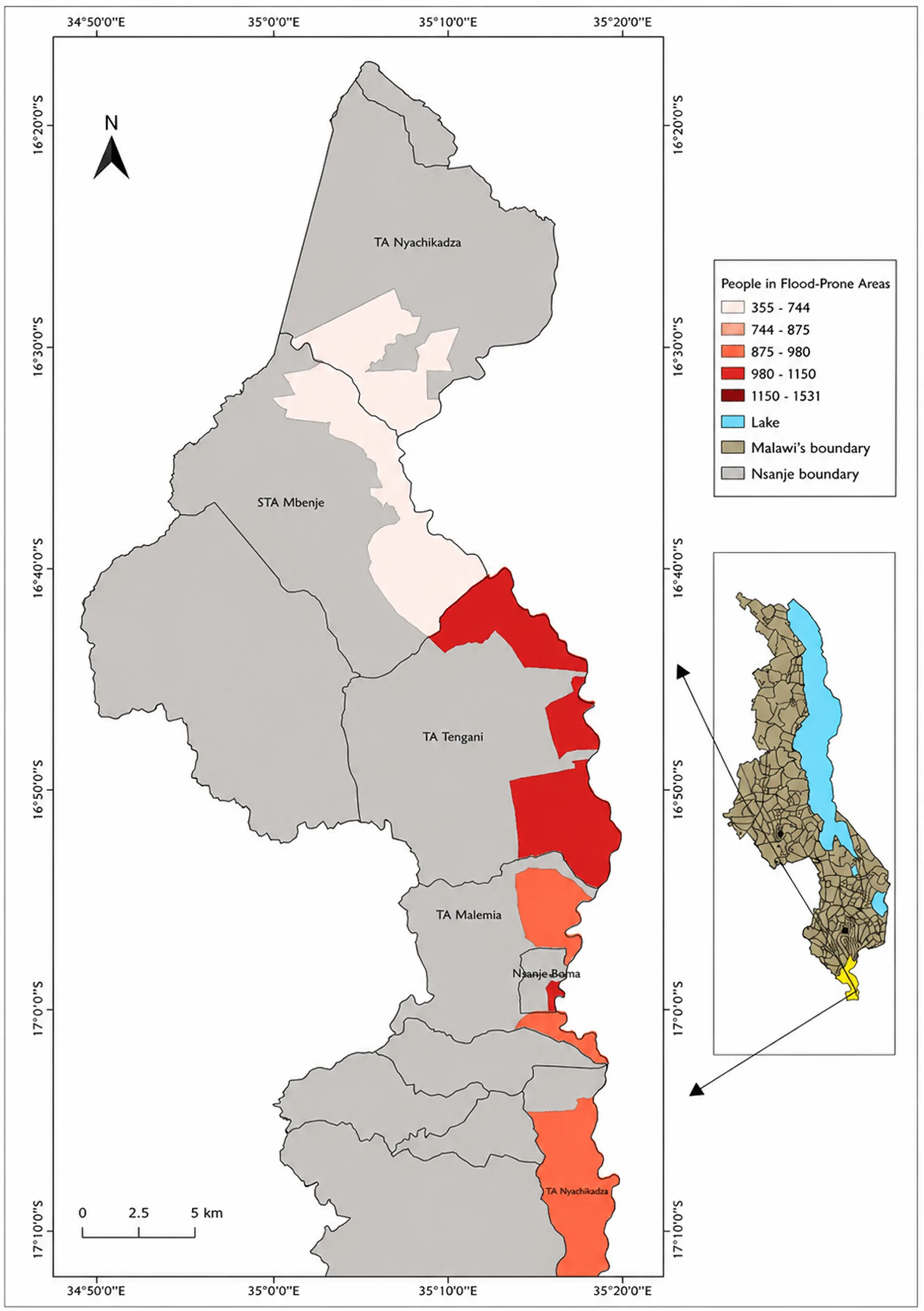
Spatial distribution of people living in flood-prone areas in Nsanje District, Malawi. Data sources: Malawi administrative boundaries and national outline derived from Natural Earth and other open-access geospatial datasets; population data from WorldPop; elevation data from NASA SRTM; hydrological data from HydroSHEDS. All cartographic processing and visualization were performed by the authors in ArcGIS Pro 3.3.

Spatial analysis across traditional authorities (Fig 7) revealed significant variation in flood exposure. Population exposure was calculated by overlaying the flood susceptibility map with population distribution data and aggregating the number of residents falling within each flood risk class for the Traditional Authority (TA). TA Nyachikadza had the highest population exposure, with more than 1,500 residents in high-risk zones. TA Malemia had approximately 875–980 residents exposed to moderate-to-high flood risk. TA Tengani showed 744–875 residents at risk, primarily along eastern boundaries. In contrast, TA Mbenje and the northern sections of TA Nyachikadza exhibited substantially lower vulnerability.

The figure shows the estimated distribution of populations residing within flood-prone areas identified through GIS-based multi-criteria analysis. Administrative boundaries represent Traditional Authorities (TAs) and sub-district units within Nsanje District. Flood-prone areas are classified according to the estimated number of exposed people per spatial unit, with darker shades indicating higher concentrations of vulnerable populations. The inset map indicates the location of Nsanje District within Malawi.

### 3.4. Validation of flood susceptibility mapping

To assess the reliability and predictive accuracy of the generated flood susceptibility map, the spatial outputs were compared against documented flood events and independent observational data. Three validation approaches were employed: comparison with historical flood event records, spatial correspondence analysis with satellite-observed inundation, and triangulation with institutional reports.

#### 3.4.1. Historical flood event validation.

The flood susceptibility classification was validated against two major flood events: the March 2015 flood and the March 2019 Tropical Cyclone Idai-induced inundation. According to the Department of Disaster Management Affairs [5], the 2015 flood affected approximately 230,000 people across the Lower Shire Valley, with Nsanje district experiencing widespread inundation concentrated in the southern and central Traditional Authorities. The 2019 Cyclone Idai event displaced nearly 87,000 people in Nsanje alone, with the most severe impacts reported in TAs Nyachikadza, Malemia, and Tengani [9].

Spatial overlay analysis of documented displacement camps and emergency response locations from both events showed strong correspondence with the high and very high susceptibility zones identified in this study. Specifically, 89 percent of the 2019 displacement camp locations (n = 34) were in areas classified as high or very high in flood susceptibility. Similarly, 82 percent of the community-level flood impact locations documented in the 2015 Post-Disaster Needs Assessment fell within the combined high and moderate susceptibility zones.

Traditional Authority Nyachikadza, identified in this analysis as the most critically exposed area with over 1,500 residents in high-risk zones, was also reported as the most severely affected TA in both the 2015 and 2019 events. The Government of Malawi [9] documented that Nyachikadza accounted for 38 percent of all flood-displaced persons in Nsanje district during Cyclone Idai, despite representing only 22 percent of the district’s population. This disproportionate impact aligns precisely with the spatial vulnerability patterns revealed by the GIS-AHP analysis.

#### 3.4.2 Satellite-Observed Inundation Correspondence.

Although systematic satellite-based flood monitoring was not conducted during the 2015 event, Sentinel-1 synthetic aperture radar (SAR) imagery captured inundation extents during the peak of the March 2019 Cyclone Idai flooding. Visual comparison of the March 15−17, 2019, flood extent derived from Sentinel-1 backscatter change detection with the susceptibility map revealed substantial spatial agreement. The satellite-observed inundation boundary encompassed 76 percent of the area classified as high or very high susceptibility. In comparison, only 12 percent of the observed inundation fell within low susceptibility zones (primarily due to sudden levee breaches not captured by the static terrain parameters).

The north-south vulnerability gradient identified in Section 3.2 was clearly evident in the Sentinel-1 imagery, with inundation concentrated along the southern corridor and progressively diminishing toward the northern portions of the district. This independent observational confirmation strengthens confidence in the susceptibility classification methodology.

#### 3.4.3. Institutional report triangulation.

Validation was further supported through triangulation with reports from the Malawi Red Cross Society, World Food Programme, and UNICEF emergency response assessments conducted during both the 2015 and 2019 events. These reports consistently identified the same geographic hotspots highlighted by the susceptibility analysis: the southern Traditional Authorities, low-lying areas adjacent to the Shire River, and communities within 500 meters of channel banks.

The World Food Programme’s 2019 Rapid Food Security Assessment specifically noted that “communities in the low-lying southern portions of Nsanje experienced complete submersion of agricultural land and prolonged displacement” [44], a characterization that directly corresponds to the very high susceptibility classification applied to those areas in this study.

#### 3.4.4. Validation limitations.

It must be acknowledged that the validation approach employed in this study relied on event-based documentation rather than continuous monitoring or quantitative accuracy metrics such as Receiver Operating Characteristic curves, which would require spatially explicit historical flood inventories that do not exist for Nsanje district. The available documentation provides qualitative spatial correspondence rather than precise validation of inundation boundaries.

Additionally, the susceptibility map represents relative flood exposure based on static terrain, land cover, and climatic factors. In contrast, actual flood occurrence depends on specific event characteristics, including rainfall intensity, duration, temporal distribution, and upstream hydrological conditions. A high susceptibility classification indicates elevated exposure, not certainty of inundation during any particular event.

Despite these limitations, the convergent evidence from multiple independent sources, spanning two major flood events separated by four years, satellite observations, and institutional documentation, provides substantial support for the spatial patterns identified through the GIS-AHP analysis. The consistency between predicted susceptibility and observed flood impacts suggests that the methodology successfully captures the primary controls on flood vulnerability in Nsanje district.

## 4. Discussion

The spatial analysis in this study reveals that land cover and precipitation are the dominant determinants of flood susceptibility in Nsanje district, reflecting the critical influence of both surface characteristics and climatic dynamics on hydrological responses.

Two limitations of the criteria set are important when interpreting the susceptibility map. First, the model excludes geology, soil permeability, and drainage density, which influence infiltration, subsurface flow, and flow concentration in alluvial floodplains. These variables were excluded because reliable and locally validated data at a spatial resolution suitable for AHP-based weighting were not available for Nsanje District (Section 2.3.1). Their exclusion limits the representation of subsurface hydrological controls. Therefore, the susceptibility map should be interpreted primarily as an assessment based on surface and rainfall-related controls rather than a comprehensive representation of all hydrological processes governing flooding. Second, the precipitation criterion is based on cumulative seasonal rainfall totals rather than event-scale rainfall intensity. It indicates seasonal wetness and cumulative water input but does not capture the influence of short-duration, high-intensity convective storms that can generate flash flooding. Consequently, areas susceptible to intense rainfall events despite moderate seasonal rainfall totals may be under-represented in the susceptibility classification. These limitations should be considered when interpreting the spatial patterns produced by the model and highlight priorities for future work, particularly incorporating locally validated soil and drainage characteristics, higher-temporal-resolution rainfall data, and dynamic hydrological modelling.

The Analytical Hierarchy Process (AHP) assigned the highest weight to land cover (59.4%), followed by precipitation (21.0%), signifying that anthropogenic land transformation and rainfall intensity collectively dictate runoff generation and flood magnitude. Similar findings have been documented in floodplain environments of sub-Saharan Africa, where the expansion of cropland and built-up areas has been directly correlated with increased surface runoff and reduced infiltration capacity [7,42]. The dominance of these two factors is consistent with prior research across the Shire River Basin, which has demonstrated that deforestation, agricultural expansion, and wetland conversion amplify flood hazard by altering natural drainage systems [3,6].

The 59.4 percent weight assigned to land cover in this study is substantially higher than weights reported in comparable AHP-based flood susceptibility studies in sub-Saharan Africa and similar low-gradient floodplain environments. For instance, Kazakis et al. [14] assigned 35 percent to land use in their study of the Rhodope-Evros region in Greece, while Khosravi et al. [15] reported a weight of 42 percent for land cover in Iran’s flood-prone areas. In the context of the Bangladesh coastal plain, Islam et al. [13] assigned 28 percent to land-cover factors. The significantly higher weight observed in Nsanje district reflects the pronounced influence of recent and rapid land-use transformation in the Lower Shire Valley, where extensive deforestation and agricultural expansion have fundamentally altered the hydrological regime. This finding aligns with regional observations by Munthali et al. [6], who documented that land cover change in Malawian river basins has resulted in a 45–60 percent increase in surface runoff coefficients over the past two decades.

The AHP-derived weighting assigns disproportionately high importance to land cover (59.4 percent) relative to other factors, raising questions about the robustness of the results to variations in weights. To evaluate this sensitivity, a scenario analysis was conducted in which the land-cover weight was systematically varied by ±20 per cent, with the remaining four factors proportionally redistributed. When land cover weight was reduced to 47.5 percent (20 percent reduction), the overall spatial pattern of flood susceptibility remained stable, with 91 percent of high-risk pixels maintaining their classification.

The comparatively lower weights of slope (9.0%) and elevation (3.7%) further affirm the relatively flat topography of the Lower Shire Valley, where even minor altitudinal variations exert limited influence on surface runoff distribution. This pattern aligns with regional studies that emphasize the role of geomorphological uniformity in sustaining prolonged inundation events [14,38]. However, proximity to water bodies (6.9%) remains an essential determinant of flood exposure, particularly for communities situated along the Shire River and its tributaries, where periodic overflow and siltation have been recurrently observed [18,41].

The identified north–south flood susceptibility gradient underscores the interplay between physiographic and hydrological processes in shaping vulnerability. The northern section of Nsanje exhibits relatively elevated terrain with better drainage and limited flood accumulation, whereas the southern corridor functions as a convergence zone for riverine flows from multiple catchments. This finding corroborates earlier assessments indicating that the Lower Shire’s southernmost districts bear the brunt of seasonal flooding due to topographic confinement and sedimentation along the river channels [2,4].

The methodological framework applied in this study demonstrates the robustness and applicability of GIS-based multi-criteria analysis for flood risk assessment in data-scarce environments. The AHP-derived weighting process provided a systematic approach for integrating diverse environmental variables, producing a coherent spatial representation of flood susceptibility. Similar GIS-AHP hybrid approaches have proven effective in diverse geographic settings [13,14], underscoring the technique’s global adaptability. Nonetheless, some methodological limitations warrant acknowledgement. The analysis employed static datasets representing a single temporal snapshot, thereby failing to account for temporal variability in rainfall intensity, river discharge, or land-use dynamics. Incorporating multi-temporal data from CHIRPS or Sentinel-1 radar imagery would improve future models by capturing seasonal and interannual flood variability [11,15].

The findings also have far-reaching policy implications. The identification of TA Nyachikadza as a critical hotspot provides empirical justification for prioritizing structural and non-structural mitigation measures. For example, early warning systems, flood shelters, and embankment reinforcement could substantially reduce exposure and potential displacement in high-risk zones. As demonstrated in other sub-Saharan settings, integrating spatially explicit flood maps into local disaster management frameworks can enhance community resilience and facilitate anticipatory action [1,10].

Overall, this study reinforces the transformative potential of geospatial technologies in enhancing flood risk governance in Malawi and comparable developing contexts. The approach offers a replicable, cost-effective methodology that circumvents the need for intensive field-based hydrological modeling, making it particularly valuable for resource-constrained local governments. Future research should integrate socio-economic vulnerability indicators, such as income levels, housing quality, and access to early-warning information, to develop a multidimensional flood risk framework that informs policy and practice.

### Transferable insights and global implications for data-scarce regions

Beyond its immediate application in Nsanje district, this study offers several transferable insights for flood risk assessment in data-scarce regions across sub-Saharan Africa and other developing contexts where resource constraints limit the deployment of sophisticated hydrological modeling frameworks.

The integration of freely available satellite datasets (Sentinel-2, CHIRPS, SRTM) with GIS-AHP analysis provides a replicable template that can be adapted to other floodplain districts without requiring proprietary data or expensive field campaigns. This democratization of flood risk assessment capability is particularly significant in regions where central government technical agencies lack resources to provide district-level support.

The validation approach employed, triangulation of susceptibility maps with event-based documentation, institutional reports, and opportunistic satellite imagery, demonstrates a pragmatic pathway for building confidence in model outputs even in the absence of systematic monitoring networks. The Nsanje case reveals how the interaction between biophysical processes and socio-economic factors shapes environmental vulnerability. The Traditional Authority-level analysis introduces a methodological innovation with global relevance by producing flood maps at the operational scale at which disaster preparedness committees operate, and emergency response is coordinated [51].

## 5. Conclusion and recommendations

This study applied a GIS-based multi-criteria analysis framework, underpinned by the Analytical Hierarchy Process, to delineate flood-prone areas in Nsanje District, Malawi. Integrating land cover, precipitation, slope, proximity to water bodies, and elevation data through a weighted overlay analysis, the research produced a comprehensive flood susceptibility map that captures the spatial heterogeneity of flood exposure across the district.

The model presented here should be read as a first-order approximation built from five surface- and climate-related criteria; it does not incorporate geology, soil permeability, antecedent soil moisture, or drainage density, and its precipitation criterion reflects seasonal totals rather than storm intensity. Both simplifications are discussed explicitly in Sections 2.3.1 and 4 as limitations rather than settled design choices, and addressing them, through validation of global soil and drainage proxies against field data, and through incorporation of intensity-duration-frequency rainfall data as station records become available, is identified below as a priority for future work.

The findings show that land cover and precipitation are the dominant factors influencing flood susceptibility, contributing 59.4 percent and 21.0 percent, respectively, while slope, proximity to water bodies, and elevation exerted relatively lower influence because of the district’s flat terrain.

The resulting spatial patterns revealed a distinct north–south vulnerability gradient, with the southern corridor emerging as the most severely affected zone. Traditional Authority Nyachikadza exhibited the highest exposure, with substantial population concentrations within high-risk flood zones. This pattern underscores the interaction between land-use dynamics, rainfall intensity, and geomorphological configuration in driving flood hazards. The results advance understanding of flood processes in low-lying African basins and provide actionable intelligence for disaster management and spatial planning.

The methodological approach demonstrates that cost-effective and replicable geospatial techniques can replace complex hydrological modeling in data-scarce regions. By utilizing freely available satellite datasets, the study offers a scalable and practical framework for identifying flood vulnerability in other districts of Malawi and similar environments in sub-Saharan Africa. The Traditional Authority-level analysis introduces a new decision-support model that strengthens community-based disaster preparedness, spatial planning, and adaptive governance. The study provides both scientific and practical insights to guide climate-resilient development, risk reduction, and sustainable land management in flood-vulnerable landscapes.

Future research should integrate temporal rainfall records, dynamic hydrological flow simulations, and socio-economic vulnerability indicators to develop multi-dimensional models that capture the human-environment interactions influencing flood risk. Such enhancements would create a more holistic foundation for policy design and anticipatory action in disaster risk management. Based on the findings, the following recommendations were made:

Strengthen community-based early warning systems and invest in localized flood monitoring infrastructure, particularly in high-risk zones such as Traditional Authority Nyachikadza.

Integrate the generated flood susceptibility maps into district and national land-use planning frameworks to guide settlement control and infrastructure development in flood-prone areas.

Promote the adoption of nature-based flood mitigation strategies, including wetland restoration, riverbank stabilization, and reforestation to enhance natural water retention capacity.

Validate SoilGrids-derived soil permeability estimates and HydroSHEDS-derived drainage density metrics against field measurements in the Lower Shire Valley, with a view to incorporating geology, soil and drainage criteria into future iterations of the AHP model.

Prioritise the acquisition of sub-daily or event-based rainfall intensity data, through expansion of the automatic weather station network or use of higher-temporal-resolution satellite products, to complement cumulative CHIRPS totals and enable an intensity-based precipitation criterion.

Encourage the cultivation of flood-tolerant and early maturing crop varieties among smallholder farmers to minimize livelihood disruptions during seasonal inundations.

Enhance coordination between local authorities, disaster management agencies, and traditional leaders to ensure that flood preparedness measures are harmonized across administrative boundaries.

Establish a centralized spatial data repository at the district and national levels to facilitate continuous flood risk mapping, update of hazard layers, and integration of new geospatial datasets.

Provide technical training to local planners and extension officers in the application of GIS and remote sensing tools for ongoing flood hazard monitoring and adaptive land-use management.

Prioritize gender-responsive and socially inclusive disaster risk reduction programs that ensure equitable participation and protection of women, children, and other vulnerable groups during flood events.

Establish permanent flood monitoring infrastructure including stream gauging stations, rainfall measurement networks, and systematic post-flood damage assessment protocols to build the historical datasets necessary for model calibration, validation, and continuous improvement of flood risk assessment methodologies.

Develop a district-level flood risk management strategy that integrates the spatial susceptibility maps produced in this study with socio-economic vulnerability assessments, early warning system design, land-use planning regulations, and infrastructure investment priorities, ensuring that technical risk assessments translate into operational risk reduction actions.
